# Development of Constitutive Relationship for Thermomechanical Processing of FeCrAl Alloy to Predict Hot Deformation Behavior

**DOI:** 10.3390/ma18133007

**Published:** 2025-06-25

**Authors:** Chuan Li, Shuang Chen, Shiyu Du, Juhong Yu, Yiming Zhang

**Affiliations:** 1 School of Mechanical and Electrical Engineering, Jiangxi University of Science and Technology, Ganzhou 341000, China or lichuan@nimte.ac.cn (C.L.); chenshuang826@126.com (S.C.); 2 Zhejiang Key Laboratory of Data-Driven High-Safety Energy Materials and Applications, Ningbo Key Laboratory of Special Energy Materialss and Chemistry, Ningbo Institute of Materials Technology and Engineering, Chinese Academy of Sciences, Ningbo 315201, China; 3 Qianwan Institute of CNITECH, Ningbo 315336, China; 4 School of Materials Science and Engineering, China University of Petroleum (East China), Qingdao 266580, China; 20220133@upc.edu.cn

**Keywords:** hot deformation behavior, constitutive model, artificial neural network

## Abstract

Numerical simulation is a vital tool in the development of FeCrAl alloy cladding tubes, with its reliability closely tied to the predictive accuracy of the thermal deformation constitutive model used. In this study, hot compression tests on 0Cr23Al5 alloy were conducted using a Gleeble-3800 thermal compression testing machine (Dynamic Systems Inc., located in Albany, NY, USA), across a temperature range of 850–1050 °C and a strain rate range of 0.1–10 s^−1^. Based on the data obtained, both the Arrhenius constitutive model and the artificial neural network (ANN) model were developed. The ANN model demonstrated significantly superior predictive accuracy, with an average absolute relative error (AARE) of only 0.70% and a root mean square error (RMSE) of 1.99 MPa, compared to the Arrhenius model (AARE of 4.30% and RMSE of 14.47 MPa). Further validation via the VUHARD user subroutine in ABAQUS revealed that the ANN model has good applicability and reliability in numerical simulations, with its predicted flow stress showing high consistency with the experimental data. The ANN model developed in this study can effectively predict the rheological stress of FeCrAl alloys during hot deformation. It provides methodological support for high-fidelity constitutive modeling of the flow stress of FeCrAl alloys and offers a reliable constitutive model for simulating the thermomechanical load response behavior of FeCrAl alloys.

## 1. Introduction

The Fukushima nuclear accident shed light on a critical drawback of zirconium alloys, which are prone to violent oxidation reactions with high-temperature steam. This leads to the release of substantial quantities of hydrogen and heat. Subsequently, there emerged an acute demand within the nuclear energy sector for the development of accident-tolerant fuel cladding materials. The aim is to enhance the safety margin of nuclear reactors operating under extreme conditions [1,2]. Among various candidates, FeCrAl alloys stand out. They possess remarkable high-temperature mechanical properties, corrosion resistance, and radiation tolerance, which make them the most promising short-term alternative to zirconium alloy cladding [3,4]. Nevertheless, FeCrAl alloys present a notable challenge due to their large neutron absorption cross-section. This necessitates either an increase in fuel enrichment or a reduction in cladding thickness to offset the neutron economy losses. As increasing fuel enrichment can have implications for the fuel cycle, the common approach is to decrease the thickness of FeCrAl alloy cladding to 0.3 mm. This, in turn, heightens the difficulty associated with the precision forming and processing of FeCrAl alloys [5]. Presently, the forming of FeCrAl alloy tubes primarily involves processes such as drawing and pilger rolling. These processes are typically preceded by steps like forging, extrusion, and rolling [6]. The presence of multiple elements in FeCrAl alloys can lead to the formation of refractory compound precipitates. As a result, in the as-forged state, these alloys exhibit high strength but low ductility, which poses unfavorable conditions for subsequent rolling operations [7]. Yamamoto et al. highlighted that the key to addressing issues such as grain coarsening, uneven wall thickness, and fracture after multi-pass drawing lies in optimizing the thermal processing parameters by selecting appropriate annealing temperatures and times for FeCrAl alloys of different compositions [8,9]. During hot working, deformation mechanisms like work hardening (WH), dynamic recovery (DRV), and dynamic recrystallization (DRX) can individually or collectively exert an influence. They affect the microstructure and ultimate mechanical properties of FeCrAl alloys [10]. Therefore, gaining an understanding of how the flow stress depends on key processing parameters (such as strain, strain rate, and deformation temperature) during the hot working of FeCrAl alloys is of vital importance. It is crucial for fully unlocking the potential of FeCrAl alloys as alternative fuel cladding materials [11,12].

Constitutive models, which provide a quantitative characterization of the true stress–strain response during deformation, represent an effective approach for analyzing the high-temperature flow behavior of metals [13,14]. In recent years, numerous constitutive equations have been proposed by researchers to describe the high-temperature deformation of metallic materials. Among these, the Arrhenius constitutive model stands out as one of the most representative ones [15]. However, the conventional Arrhenius constitutive model falls short in accuracy, as it neglects strain effects. Shi enhanced the predictive capability of the model by incorporating material strain and temperature parameters [16]. Since then, the model has been widely used to characterize the thermal deformation of various metals and alloys, such as austenitic stainless steel, high-temperature alloys, aluminum, magnesium, and titanium alloys [17,18,19,20]. Several researchers have employed a strain-compensated Arrhenius constitutive model to develop constitutive models for FeCrAl alloys of different compositions across various temperature and strain rate ranges [21,22,23,24]. In these studies, the average absolute relative error (AARE) between the calculated and experimental values generally fell within 4~8%. This inaccuracy is primarily attributed to the fact that the Arrhenius constitutive model is typically developed for specific deformation mechanisms. Consequently, it lacks continuity in describing the transition between different deformation mechanisms. Moreover, it fails to comprehensively account for the coupled effects of multiple deformation mechanisms on the high-temperature flow stress during thermal deformation, resulting in insufficient predictive accuracy [25]. Accurate numerical simulation of plastic deformation requires a thorough understanding of the complex relationship between material flow stress and deformation parameters. Therefore, to accurately describe the thermal deformation of FeCrAl alloys, it is imperative to develop a high-precision constitutive model.

With the advancement of computer technology, artificial neural networks (ANN) have emerged as a powerful tool for developing material constitutive models, thanks to their remarkable nonlinear data processing capabilities [26]. Unlike phenomenological models, which require presupposing a mathematical–physical expression to determine material parameters in constitutive models, ANN models directly learn from data. This data-driven approach enables ANN models to achieve higher efficiency and accuracy in establishing material constitutive models. A number of studies have successfully applied ANN models to construct flow stress models for high-entropy alloys [27] and stainless steels [28,29,30]. Furthermore, comparisons of their predictive performance with that of the Arrhenius constitutive model in the hyperbolic sine function form have been conducted. The results indicate that there is an extremely high degree of consistency between the predicted stress values generated by ANN models and the experimentally measured values. This highlights the effectiveness of utilizing artificial neural networks to model the flow behavior of metals and alloy materials.

Given the limitations of experimental cost and testing duration, numerical simulation has emerged as a crucial method for analyzing the performance of cladding tubes. The accuracy of the input material’s stress–strain data plays a vital role in determining the reliability of the simulation results. For other large-sized structural components, the prediction errors of phenomenological constitutive models, such as the Arrhenius constitutive model, might be within an acceptable range. Nevertheless, due to the small size of cladding tubes, even minor errors can be easily amplified, which may severely compromise the reliability of the simulation results. Therefore, this study aims to develop a FeCrAl flow stress model with high predictive accuracy. Based on the data obtained from hot compression experiments, both the Arrhenius constitutive model and the artificial neural network (ANN) model were constructed. Their performance in reproducing the hot deformation behavior was compared in terms of correlation coefficient, mean absolute error, and root mean square error. Furthermore, to verify the applicability of the developed high-fidelity model in numerical simulation, the ANN constitutive model was incorporated into the finite-element code via the VUHARD subroutine. Its predictions were then compared with the actual deformation behavior of the cladding tube observed in hot compression experiments. This study aims to provide a reliable constitutive foundation for numerical simulation tasks that require high-accuracy, high-temperature stress–strain data. Such tasks include the processing and preparation, structural optimization, and impact analysis of FeCrAl alloy cladding tubes. At present, we are utilizing the developed constitutive model to investigate the thermos-mechanical load response behavior assessment of structures optimized by the topology optimization method.

## 2. Materials and Methods

The experimental material was a cast FeCrAl alloy ingot with the nominal chemical composition detailed in Table 1 (in wt.%), provided by Shenzhen Zhonghang Special Alloy Co., Ltd. (Shenzhen, Guangdong Province, China).

**Figure 1:**
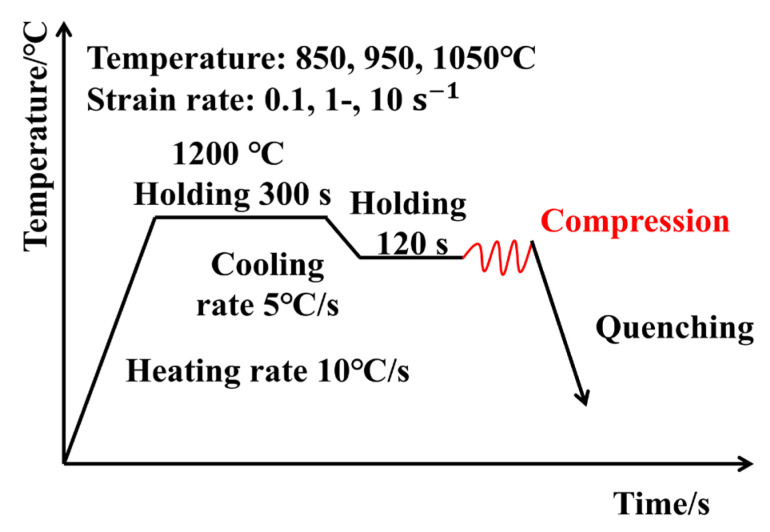
Schematic diagram of the deformation process.

Cylindrical specimens, measuring 10 mm in diameter and 15 mm in height, were prepared via wire-cut electrical discharge machining. To minimize friction during compression, graphite sheets were placed between the punch and the specimen. All tests were carried out in an argon atmosphere to prevent specimen oxidation during heating and deformation, thereby ensuring experimental accuracy. A schematic illustration of the hot compression process is presented in Figure 1. The specimens were first heated to 1200 °C at a rate of 10 °C/s and held at this temperature for 300 s. Subsequently, they were cooled to the target deformation temperatures (850 °C, 950 °C, and 1050 °C) at 5 °C/s, with a 120 s dwell at each target temperature to ensure uniform temperature distribution within the specimen. At each temperature, isothermal compression was performed at strain rates of 0.1 s^−1^, 1 s^−1^, and 10 s^−1^ until the specimen height was reduced to 50% of its initial height, achieving a true strain of approximately 0.7.

## 3. Results and Discussion

### 3.1. Flow Stress Curves

**Figure 2:**
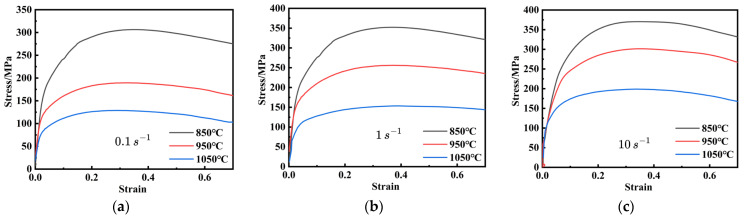
The curves of true stress-strain of FeCrAl alloy at the temperatures of 850 °C–1050 °C at the strain rates of: (**a**) 0.1 s^−1^; (**b**) 1 s^−1^; (**c**) 10 s^−1^.

True stress–strain curves are crucial for reflecting the intrinsic relationship between flow stress and deformation parameters during hot deformation. Figure 2 presents the true stress–strain curves of FeCrAl alloys under various strain rates and deformation temperatures. These curves display similar trends: flow stress decreases with lower strain rates and higher temperatures. This can be attributed to the fact that high temperatures enhance dislocation and grain-boundary mobility, thereby reducing flow stress [31].

In the initial deformation stage, the curve exhibits a rapid increase with the rise of strain, predominantly due to work hardening (WH), which is characterized by an increase in dislocation density. It is worth noting that, although dynamic recovery (DRV) commences almost immediately at the start of plastic deformation and work-hardening competition, the effects of DRV and dynamic recrystallization (DRX) become more significant as deformation proceeds. This is evident from the gradual decrease in flow stress [32,33]. Moreover, at a strain rate of 1 s^−1^ and a temperature of 1050 °C, the flow stress rapidly rises to a peak, then stabilizes after a gradual decrease, indicating a balance between WH, DRV, and DRX. These findings indicate that flow stress in FeCrAl alloys has a highly nonlinear relationship with strain, the strain rate, and the temperature during hot deformation.

### 3.2. Improved Arrhenius-Type Constitutive Model

Constitutive models, offering a mathematical description of hot deformation, serve as a theoretical foundation for numerical simulation analysis [34]. In this study, we utilize the strain-compensated Arrhenius constitutive model proposed by Lin [35], which takes the hyperbolic sine form as follows:

(1) $$\begin{array}{c} \dot{\varepsilon }=A_{1}\sigma^{n_{1}}\exp\left(-\frac{Q}{RT}\right)\left(\alpha \sigma <0.8\right) \end{array}$$

(2) $$\begin{array}{c} \dot{\varepsilon }=A_{2}\exp\left(\beta \sigma \right)\exp\left(-\frac{Q}{RT}\right)\left(\alpha \sigma >1.2\right) \end{array}$$

(3) $$\begin{array}{c} \dot{\varepsilon }=A[\sinh\left(\alpha \sigma \right){]}^{n}\exp\left(-\frac{Q}{RT}\right)\left(for\;all\right) \end{array}$$

where the $\dot{\varepsilon }$ symbol represents the strain rate, *σ* represents the flow stress, and *Q* (J/mol) represents the thermal activation energy of deformation, which characterizes the energy barrier that the material must overcome during hot deformation. R is the universal gas constant, with a value of 8.3145 J/(mol K). *T* denotes the absolute temperature in Kelvin (K). And *α* (MPa^−1^) is an adjustable constant, reflecting the influence of stress level on deformation. And *n* is the stress exponent, indicating the degree to which stress affects the strain rate. *A* is the structural factor, which reflects the influence of the material’s structural characteristics under specific deformation conditions on the deformation behavior.

Taking the logarithm of both sides of Equations (1) and (2) yields the following equations:

(4) $$\begin{array}{c} \begin{array}{c} \ln\dot{\varepsilon }=\ln A_{1}+n_{1}\ln\sigma \end{array} \end{array}$$

(5) $$\begin{array}{c} \ln\dot{\varepsilon }=\ln A_{2}+\beta \sigma \end{array}$$

**Figure 3:**
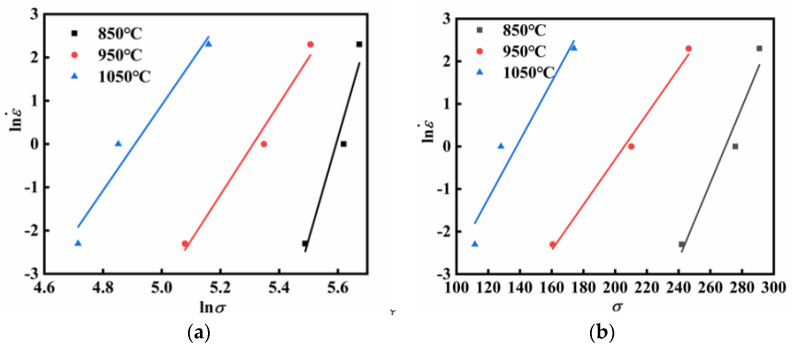
The linear relationship fitting at peak stress: (**a**) ln$\dot{\varepsilon }$-ln*σ*; (**b**) ln$\dot{\varepsilon }$-*σ*.

By utilizing Equations (4) and (5) and substituting the peak stress data, linear relationships between ln$\dot{\varepsilon }$ and ln*σ*, as well as between ln$\dot{\varepsilon }$ and *σ*, were established through fitting, as shown in Figure 3. The values of *n*_1_ and *β* were calculated as the reciprocal of the average slope of the fitted straight lines, resulting in *n*_1_ = 17.299 and *β* = 0.131 MPa^−1^. Additionally, *α* was calculated as *α* = *β*/*n*_1_ = 0.00757 MPa^−1^.

**Figure 4:**
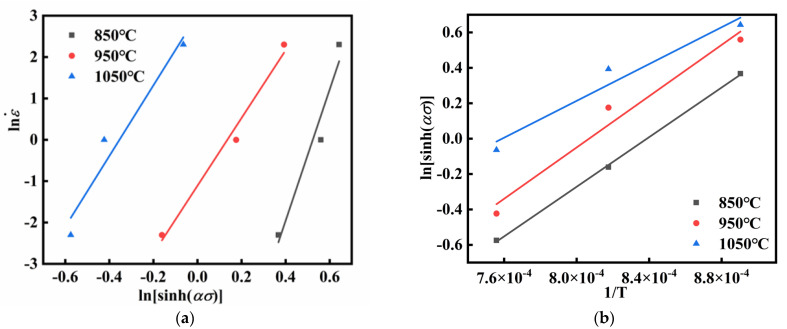
The linear relationship fitting at peak stress: (**a**) ln$\dot{\varepsilon }$-lnsinh*ασ*; (**b**) lnsinh*ασ*-1/*T*.

Taking the logarithm of both sides of Equation (3) gives Equation (6). For a constant temperature, taking the partial derivative of Equation (6) leads to Equation (7). By combining Equations (6) and (7), the expression for n is obtained as shown in Equation (8). Through calculating the average slope of ln$\dot{\varepsilon }$-lnsinh*ασ* in Figure 4a, the value of *n* was determined to be 10.39722.

(6) $$\ln\dot{\varepsilon }=\ln A+n\ln\left[\sinh\left(\alpha \sigma \right)\right]-\frac{Q}{RT}$$

(7) $$\begin{array}{c} Q=R\times {\left\{\frac{\partial \ln\dot{\varepsilon }}{\partial \ln\left[\sinh\left(\alpha \sigma \right)\right]}\right\}}_{T}\times {\left\{\frac{\partial \ln\left[\sinh\left(\alpha \sigma \right)\right]}{\partial \left(\frac{1}{T}\right)}\right\}}_{\dot{\varepsilon }} \end{array}$$

(8) $$n=\frac{\partial \ln\dot{\varepsilon }}{\partial \ln\left[\sinh\left(\alpha \sigma \right)\right]}$$

In the Arrhenius constitutive model, the deformation activation energy signifies the energy barrier for atomic migration, encompassing the alloy’s workability and thermal deformation behavior [36]. The activation energy *Q* is formulated as in Equation (9). By determining the average slope of the fitted curve in Figure 4b for lnsinh*ασ* and (1/*T*), *Q* was calculated as 585,825.067 J/mol.

(9) $$\begin{array}{c} Q=R\times n\times \left\{\frac{\partial \ln\left[\sinh\left(\alpha \sigma \right)\right]}{\partial \left(\frac{1}{T}\right)}\right\} \end{array}$$

**Figure 5:**
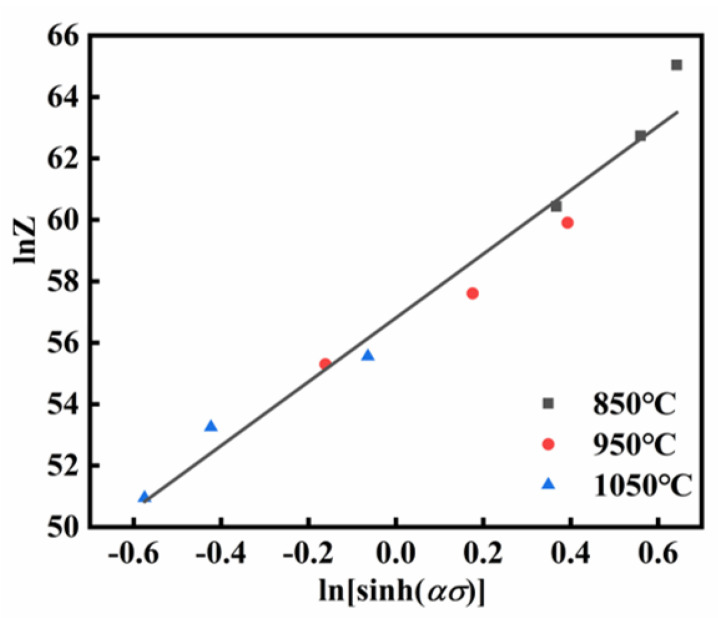
The linear relationship fitting at peak stress: ln*Z*-lnsinh*ασ*.

The *Z* parameter, as defined in Equation (10), reflects the combined influence of temperature and strain rate on a material’s thermal deformation behavior [37]. After calculating *Z*, *A* can be estimated by taking the logarithm of both sides of Equation (10), as shown in Equation (11). Figure 5 shows the fitted relationship between ln*Z* and lnsinh*ασ*. From the intercept of the fitting line, ln*A* was calculated as 56.80832.

(10) $$\begin{array}{c} Z=\dot{\varepsilon }\exp\left(\frac{Q}{RT}\right) \end{array}$$

(11) $$\begin{array}{c} \begin{array}{c} lnZ=lnA+n\ln\left[\sinh\left(\alpha \sigma \right)\right] \end{array} \end{array}$$

**Figure 6:**
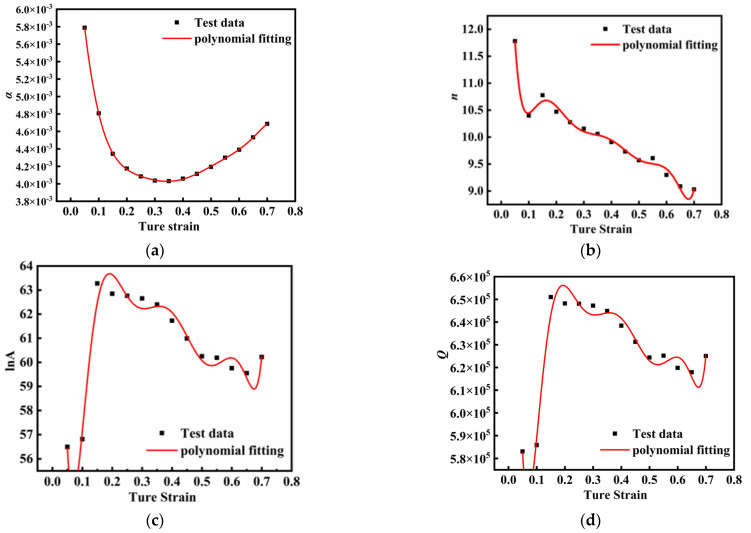
The linear fitting curves of material constants, activation energy with strain obtained through eighth-order polynomial: (**a**) α−ε; (**b**) n−ε; (**c**) lnA−ε; (**d**) Q−ε.

To account for the influence of strain on flow stress, the material constants ln*A*, *α*, *n*, and *Q* were calculated at different true strain levels, as presented in Table 2. These four parameters (α, *n*, *Q*, and *A*) exhibited nonlinear relationships with the strain variable *ε*. An increase in *Q* indicates that the deformation mechanism shifts towards a process requiring higher energy activation, such as from dislocation movement to diffusional creep. An increase in *n* suggests that the deformation mechanism shifts towards a process requiring higher stress activation, such as from dislocation slip-dominated to dynamic recrystallization-dominated. An increase in *A* means that changes in the material’s microstructure enhance the resistance to deformation, such as a reduction in grain size or an increase in dislocation density that hinders dislocation movement. An increase in *α* indicates that the material can achieve a higher strain rate at lower stress levels, which may be due to reduced hindrance to dislocation movement or the presence of mobile dislocations. Consequently, eighth-order polynomial fitting was employed to characterize the correlations between ln*A*, *α*, *n*, *Q*, and *ε*, with the fitting results illustrated in Figure 6. The corresponding equations are expressed as Equation (12). The fitting effects of the fifth-order polynomial commonly used in other literature and their comparison with the fitting effects of the eighth-order polynomial are further analyzed in Appendix A.

(12) $$\left\{\begin{array}{c} lnA=104.26532-2073.34001\varepsilon +32034.74528\varepsilon^{2}-237793.684\varepsilon^{3}+984566.6184\varepsilon^{4}-2397583.759\varepsilon^{5}+\\ 3411227.434\varepsilon^{6}-2622780.089\varepsilon^{7}+841498.2217\varepsilon^{8}\\ \alpha =0.00759-0.04555\varepsilon +0.19419\varepsilon^{2}+0.06946\varepsilon^{3}-3.54923\varepsilon^{4}+12.47521\varepsilon^{5}-23.68273\varepsilon^{6}+20.58261\varepsilon^{7}\\ -7.13679\varepsilon^{8}\\ n=22.54056-419.61037\varepsilon +5755.21639\varepsilon^{2}-40726.53084\varepsilon^{3}+164963.172\varepsilon^{4}-397810.7162\varepsilon^{5}+\\ 564350.6408\varepsilon^{6}-434496.3344\varepsilon^{7}+139972.073\varepsilon^{8}\\ Q=1059767.336-20634800\varepsilon +317954000\varepsilon^{2}-2354200000\varepsilon^{3}+9731090000\varepsilon^{4}-23675800000\varepsilon^{5}\\ +33676900000\varepsilon^{6}-25899000000\varepsilon^{7}+8314270000\varepsilon^{8} \end{array}\right.$$

**Figure 7:**
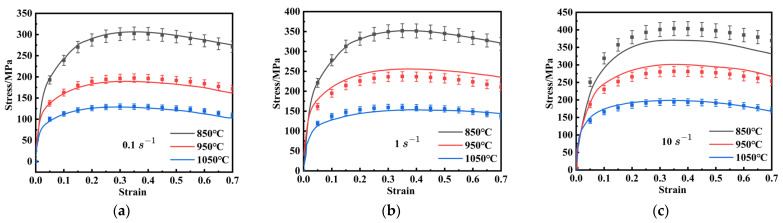
Comparison of experimental flow stress (curve) and predicted flow stress by Arrhenius constitutive model obtained through eighth-order polynomial (symbol): (**a**) 0.1 s^−1^; (**b**) 1 s^−1^; (**c**) 10 s^−1^.

Using the equations mentioned above, the flow stress at arbitrary strain levels can be calculated via Equation (3). A comparison between the stresses predicted by the Arrhenius constitutive model and the experimental measurements (Figure 7) shows consistent systematic differences over a wide range of temperatures and strain rates. This is attributed to the fact that the Arrhenius constitutive model is primarily based on empirical formulas and lacks a profound physical interpretation of the material deformation mechanisms. As a result, when confronted with complex deformation conditions, the applicability of the model and the direction for its improvement become unclear.

**Figure 8:**
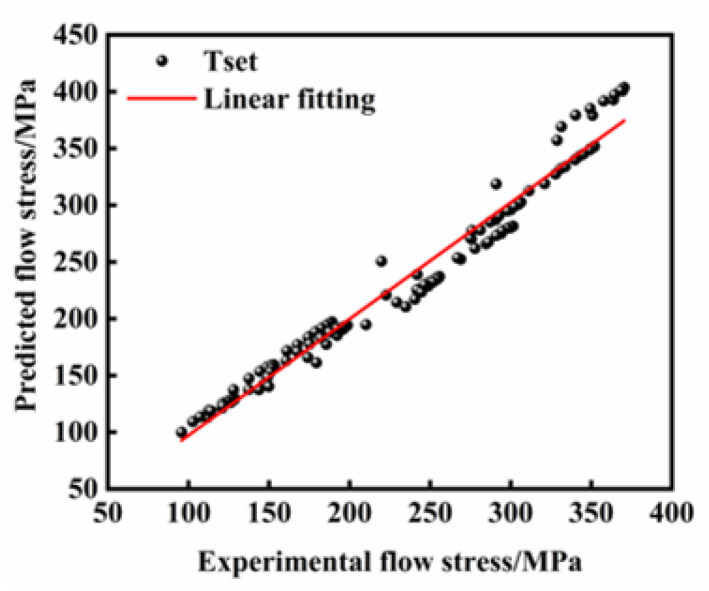
Correspondence between flow stress predictions derived from the Arrhenius constitutive model and experimentally determined flow stress values.

Figure 7 and Figure 8 show the correlation between predicted stress values from the Arrhenius constitutive model and experimentally measured stress data. Most data points cluster near the ideal fitting line, showing a general agreement between the model predictions and the experimental measurements. However, noticeable deviations occur, particularly in regions of high flow stress. These results collectively demonstrate that the Arrhenius constitutive model has inherent limitations in fully describing the flow stress behavior of FeCrAl alloys under thermomechanical deformation conditions.

### 3.3. Development of ANN Model

**Figure 9:**
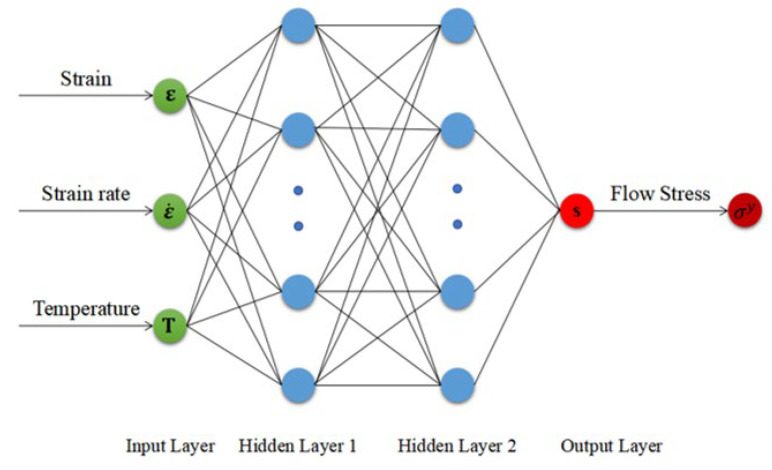
Multi-layer artificial neural network architecture.

This study utilizes a feedforward multilayer artificial neural network (ANN) model architecture, trained via the backpropagation (BP) algorithm. As shown in Figure 9, the BP-ANN neural network structure consists of three distinct layers: an input layer, hidden layers, and an output layer. Each hidden layer contains numerous simple processing units called neurons. Information propagates sequentially across the layers through interconnecting pathways or “synaptic links” [38]. The signal transmission intensity in these connections is regulated by adjustable weights linked to each neuron. By iteratively optimizing the weights and biases of individual neurons, the network learns to identify patterns within the input data.

This study highlights the importance of data normalization due to the heterogeneous units, varying ranges, and dimensionalities of strain, strain rate, deformation temperature, and flow stress. Using raw data directly would negatively impact model convergence efficiency and prediction accuracy. Given the use of sigmoid activation functions in the hidden and output layers, the three input variables were normalized to dimensionless units within the [0, 1] interval, per Equations (13)–(16). Notably, the strain rate data underwent logarithmic transformation before normalization due to its significant order-of-magnitude variations [39]. The normalized parameters were then propagated through the neural network via feedforward propagation. Finally, the output layer values were denormalized to restore the predictions to their original physical scales, enabling systematic error analysis by comparing predicted flow stress values with experimental measurements.

(13) $$\begin{array}{c} \begin{array}{c} \varepsilon =\frac{\varepsilon -\varepsilon_{min}}{\varepsilon_{max}-\varepsilon_{min}} \end{array} \end{array}$$

(14) $$\begin{array}{c} \dot{\varepsilon }=\frac{\ln\left(\frac{\dot{\varepsilon }}{{\dot{\varepsilon }}_{0}}\right)-{\ln\left(\frac{\dot{\varepsilon }}{{\dot{\varepsilon }}_{0}}\right)}_{min}}{{\ln\left(\frac{\dot{\varepsilon }}{{\dot{\varepsilon }}_{0}}\right)}_{max}-{\ln\left(\frac{\dot{\varepsilon }}{{\dot{\varepsilon }}_{0}}\right)}_{min}} \end{array}$$

(15) $$\begin{array}{c} T=\frac{T-T_{min}}{T_{max}-T_{min}} \end{array}$$

(16) $$\begin{array}{c} \begin{array}{c} \sigma^{y}=\left(\sigma_{max}-\sigma_{min}\right)\;\cdot s+\sigma_{min} \end{array} \end{array}$$

where ${\left[\;\right]}_{min}$ and ${\left[\;\right]}_{max}$ represent the minimum and maximum values at the boundary of the range for the deformation parameters (strain, strain rate, and temperature), respectively.

The ANN learning phase was numerically implemented in Python 3.11 using the TensorFlow 2.17 library. The adaptive moment estimation (ADAM) optimizer was employed to minimize the objective function [40]. Under each temperature and strain rate condition, 71 flow stress values were systematically sampled at 0.01 strain intervals within the 0~0.7 strain range. This generated a comprehensive dataset of 648 quadruple data points. The dataset was split into training and validation subsets in a 3:1 ratio. During training, input–output pairs were used to iteratively update neuronal weights and biases by minimizing the error between the predicted and target outputs. The network architecture was optimized with a 0.1 learning rate over 50,000 training iterations to ensure robust parameter convergence.

Quan et al. [41] compared the ANN architectures with single and double hidden layers, showing that the dual-hidden-layer configuration has better predictive performance. Thus, this study uses a dual-hidden-layer ANN architecture. Developing an accurate ANN model requires systematically determining the number of hidden layers and the neurons per layer. The optimal neuron count (*e*) in each hidden layer was determined using an empirical formula, expressed as Equation (17), where *n* and *m* are the neurons numbers in the input and output layers, and a is an integer from 1 to 10. This yields a hidden-layer neuron count range of 3~12 [42].

(17) $$\begin{array}{c} \begin{array}{c} e=\sqrt{n+m}+a \end{array} \end{array}$$

To evaluate the predictive performance of the Arrhenius constitutive model and the ANN model, we used the correlation coefficient (R), average absolute relative error (AARE), and root mean square error (RMSE). These metrics are defined in Equations (18)–(20). A higher R value (closer to one) shows better agreement between predicted and experimental values, while a lower AARE (closer to zero) means the sum of the errors between predicted and experimental values nears zero. Note that R only reflects the correlation degree between the experimental and predicted true stresses from both models. Accuracy is not only the decisive factor for model reliability, but also is assessed using RMSE to evaluate the deviation between the predicted values and the actual experimental data. Furthermore, the Prod index, defined in Equation (21), combines these indicators to account for absolute and relative errors, offering a more comprehensive model evaluation.

(18) $$R=\frac{\sum_{i=1}^{N}\left(\sigma_{i}^{e}-\bar{\sigma_{i}^{e}}\right)\left(\sigma_{i}^{p}-\bar{\sigma_{i}^{p}}\right)}{\sqrt{\sum_{i=1}^{N}{\left(\sigma_{i}^{e}-\bar{\sigma_{i}^{e}}\right)}^{2}\sum_{i=1}^{N}{\left(\sigma_{i}^{p}-\bar{\sigma_{i}^{p}}\right)}^{2}}}$$

(19) $$\begin{array}{c} AARE=\frac{1}{N}\;\sum_{i=1}^{N}\left|\frac{\sigma_{i}^{p}-\sigma_{i}^{e}}{\sigma_{i}^{e}}\right|\times 100\% \end{array}$$

(20) $$\begin{array}{c} RMSE=\sqrt{\frac{1}{N}\sum_{i=1}^{N}{\left(\sigma_{i}^{p}-\sigma_{i}^{e}\right)}^{2}} \end{array}$$

(21) $$\begin{array}{c} Prod=\sqrt{{RMSE}^{2}+{AARE}^{2}} \end{array}$$

where *N* is the total number of samples, $\sigma_{i}^{e}$ and $\sigma_{i}^{p}$ represent the experimental and predicted values of the true stress, $\bar{\sigma_{i}^{e}}$ and $\bar{\sigma_{i}^{p}}$ denote the mean values of the true stress for the experimental and predicted datasets.

**Figure 10:**
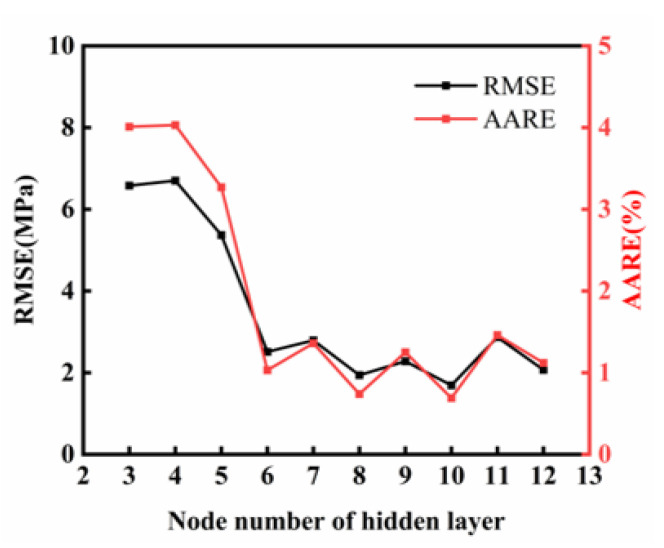
Influence of hidden nodes on the performance of ANN.

Figure 10 shows how the number of neurons in the hidden layers affects the model’s prediction accuracy. As this number increases, the model’s RMSE and AARE first decrease and then level off. This means the ANN model’s accuracy improves when the hidden layer has six or more neurons. However, beyond this point, further increasing the neurons does not significantly improve performance, indicating diminishing returns. These findings match the empirical rules for optimizing ANN architectures in regression-based predictive modeling.

We performed cyclic trials of neuron numbers from 3 to 13 to identify the optimal number of neurons in the hidden layers. Table 3 presents four ANN architectures that balance computational cost and predictive accuracy. Although the neural network’s internal variable n_v_ increases with neuron count, the training time t remains about 80 min with no significant changes. As expected, the models with more neurons show better predictive capabilities for the material’s nonlinear behavior. However, when the neuron count per layer reaches 3-12-8-1, the AARE decreases while the other three evaluation metrics worsen, possibly due to overfitting from the ANN’s high complexity. An analysis of the four evaluation metrics shows that the 3-11-6-1 ANN architecture has the smallest deviation between the predicted and experimental flow stress values. It is, thus, chosen for the finite-element numerical simulation of cylindrical thermal compression in the following section.

**Figure 11:**
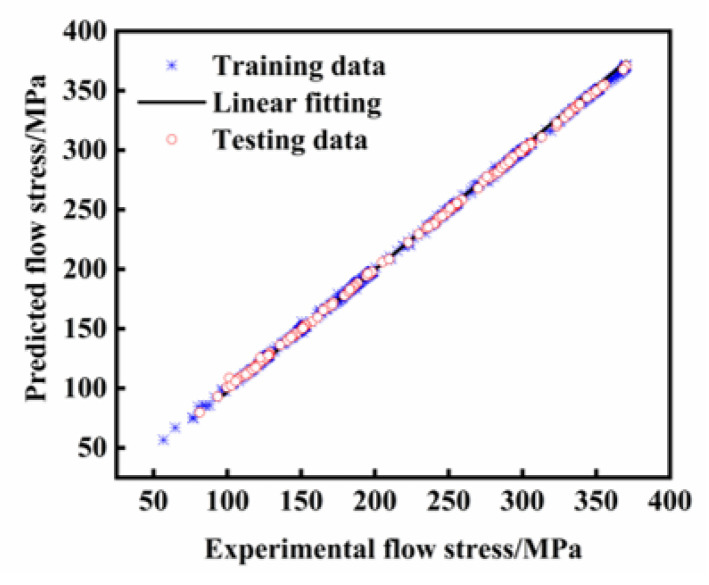
Correlation between predicted and true stress values for training data and test data.

As illustrated in Figure 11, the abscissa represents experimentally determined true stress values obtained through hot compression tests, while the ordinate displays predicted stress values, with the data points closely distributed along the optimal fitting line, demonstrating superior predictive capability. The Rs for both the training and testing datasets were calculated as 0.9936 and 0.9978, respectively. Furthermore, the AARE values derived from the training and testing sets reached 0.37% and 0.39%, accompanied by the RMSE magnitudes of 0.9634 MPa and 1.0481 MPa, respectively. The minimal discrepancies observed in both operational modes substantiate the ANN model’s exceptional predictive accuracy during both the parameter optimization and validation phases.

**Figure 12:**
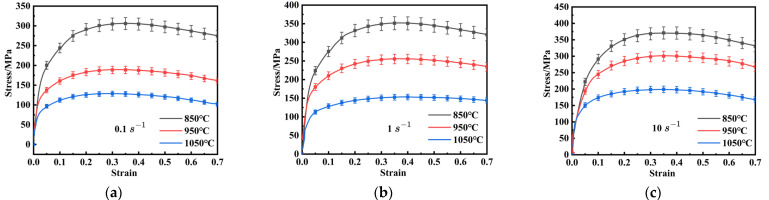
Comparison of experimental flow stress (curve) and predicted flow stress by ANN model (symbol): (**a**) 0.1 s^−1^; (**b**) 1 s^−1^; (**c**) 10 s^−1^.

The trained ANN model demonstrates competent applicability in predicting the thermomechanical behavior of FeCrAl alloys. Figure 12 presents a comparative analysis between the ANN-predicted flow stress values and the experimentally acquired data during hot compression testing. Notably, the ANN predictions exhibit remarkable congruence with experimental measurements across the entire deformation parameter spectrum, with predicted values faithfully replicating the evolutionary trends observed in the experimental data.

As quantitatively validated in Table 4, the ANN model achieves superior predictive accuracy compared to the Arrhenius constitutive model: The R and AARE for the ANN model reach 0.9995 and 0.70%, respectively, whereas the corresponding values for the Arrhenius constitutive model register at 0.9849 and 4.30%. Furthermore, critical evaluation through RMSE metrics reveals distinct performance differentials, with RMSE magnitudes of 1.99 MPa and 14.47 MPa for the ANN and Arrhenius constitutive models, respectively. These computational outcomes substantiate that the Arrhenius constitutive model manifests significantly greater prediction deviations, while the ANN architecture maintains consistently distributed predictive precision under diverse deformation conditions.

**Figure 13:**
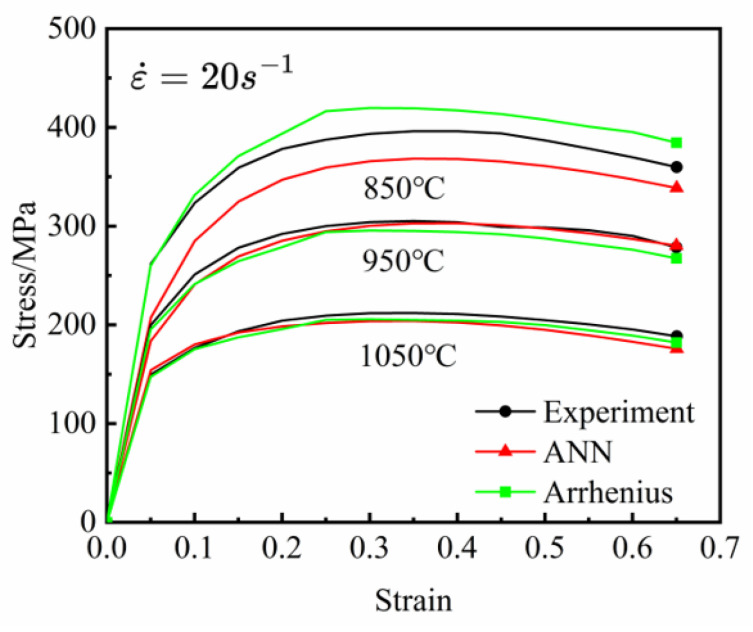
Comparison of predicted stress value of the Arrhenius constitutive model and ANN model with experimental values at 20 s^−1^ and different temperatures.

Figure 13 presents a comparative analysis of the experimental stress–strain relationships and the predictive outputs of the ANN and Arrhenius constitutive models under a newly implemented strain rate of 20 s^−1^ at three distinct deformation temperatures (850 °C, 950 °C, and 1050 °C). It can be observed that both the ANN and Arrhenius models exhibit consistent trends with the experimental values. Specifically, the ANN model demonstrates exceptional predictive tracking capability at 950 °C, with an AARE and RMSE of 1.95% and 6.5738 MPa, respectively, which are significantly better than the corresponding metrics of the Arrhenius constitutive model (3.59% and 10.6655 MPa). However, at 850 °C, particularly in the low-strain region, the prediction error of the ANN model is greater than that of the Arrhenius model. Under low-temperature conditions, the mechanisms of work hardening and dynamic softening in the material are typically less pronounced than at high temperatures, resulting in a more complex and highly nonlinear stress–strain relationship [43]. The Arrhenius model, which is based on thermodynamic principles, is capable of effectively describing the deformation behavior of the material at low temperatures. In contrast, the performance of the ANN model is highly dependent upon the quality and quantity of the training data. In the low-temperature and low-strain region, the experimental data are more susceptible to noise and uncertainties, which restrict the ANN model’s ability to learn the stress–strain relationship in this region, thereby leading to insufficient generalization capability of the ANN model in the low-temperature domain.

To address the insufficient generalization capability of the ANN model under thermal deformation conditions beyond the training data, it is recommended to incorporate experimental data from more strain rates and deformation temperatures. To establish a quantitative relationship between the microstructural characteristics and the macroscopic mechanical behavior, and to provide deeper physical insights for the model, one can use physical models to offer prior knowledge and constraints. This approach compensates for the shortcomings of data-driven models in physical interpretability and generalization capability, thereby creating more robust and adaptive predictive models. The ANN model developed in this paper is solely based on isothermal compression test data. To truly apply it to numerical simulations under more complex conditions, it is necessary to further develop datasets under multi-condition or multi-physical-field scenarios. This will provide reliable support for the development of FeCrAl alloy cladding tubes.

### 3.4. Compression Test Simulation and Verification

The effectiveness of the ANN model in reproducing the behavior of hot compression experiments has been validated, but its applicability to numerical simulation still needs further assessment. Therefore, this study conducted hot compression tests in the ABAQUS Explicit finite-element code to compare and analyze the predicted data of the ANN model with the numerical results of the hot compression experimental data. The ABAQUS/Explicit finite-element code does not directly provide a hyperbolic sine form of the constitutive model, so the VUHARD user subroutine is needed to implement it (The specific steps for deriving the function of flow stress with respect to the three inputs and their respective derivatives through the internal weights and biases of the neural network can be found in Appendix B) [44]. Through this subroutine, users can program the three derivatives of flow stress with respect to strain, strain rate, and deformation temperature as functions of the model input data, thereby enabling the customized simulation of complex constitutive relationships.

**Figure 14:**
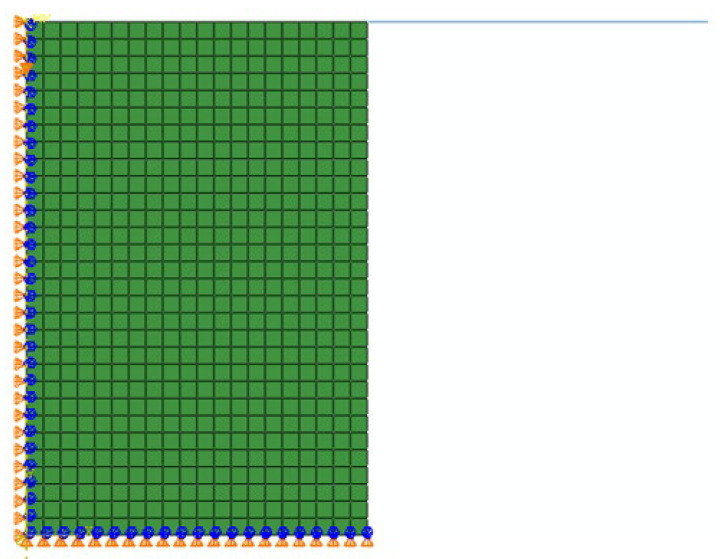
Finite-element model of the compression test.

In numerical simulation, the material used is the FeCrAl alloy introduced in Section 2. To reduce the computational cost, this study employed an axisymmetric half-model of the hot compression test specimen for simulation, the geometry of which is shown in Figure 14. The finite-element model was discretized with a mesh size of 0.25 mm, resulting in a mesh comprising 600 CAX4R elements. These elements are four-node axisymmetric bilinear quadrilaterals that employ reduced integration and hourglass control techniques. Specifically, the model is divided into 30 elements in the vertical direction and 20 elements in the radial direction. During the compression process, a total displacement of 3.75 mm was applied along the vertical axis of the specimen by the top crossbeam of the specimen. When applying the axial displacement, the radial displacement of the top surface of the specimen was kept free, while the radial displacement of the bottom surface was kept free, and the axial displacement was kept at zero. The cylindrical part of the specimen was located between two rigid surfaces, and the friction coefficient at the contact surface was set to 0.2. To further reduce the simulation time, an explicit integration scheme was adopted in this study, with a total simulation time of 3.5 s. The VUHARD user subroutine was compiled using oneAPI2022 and linked with the ABAQUS/Explicit executable file. The finite-element calculation was performed on a server running the Ubuntu 20.04 64-bit operating system, which is equipped with 16 GB of RAM and an Intel i7-12700 h processor, featuring 12 cores and 20 threads.

**Figure 15:**
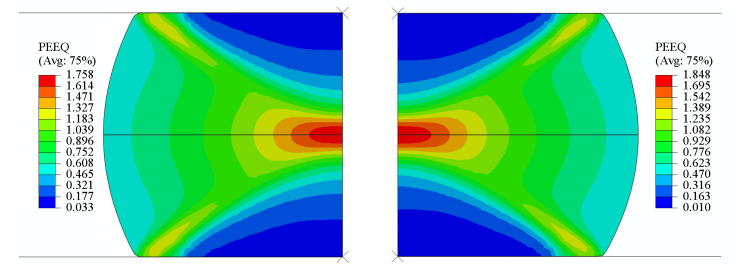
Equivalent plastic strain *ε^p^* contour plot for the compression of a cylinder using ANN flow laws (**left** side) and experimental data (**right** side).

**Figure 16:**
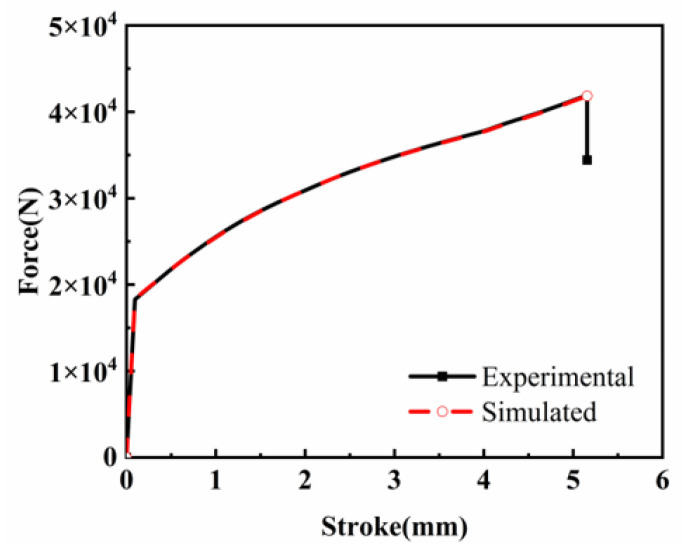
Comparison of head load–displacement curves.

Taking a strain rate of 1 s^−1^ and a deformation temperature of 850 °C as an example, Figure 15 shows the equivalent strain distribution after hot compression simulations using both experimental data and ANN model predictions. The load–stroke curves were extracted from the finite-element calculation results database, as illustrated in Figure 16. It can be observed that the spatial distribution of the equivalent plastic strain in the ANN simulation results exhibits high consistency with the experimental data, confirming the reliability of the ANN model in the flow stress identification stage.

These findings demonstrate that the ANN model developed in this study can not only effectively predict the flow stress of FeCrAl alloy during hot deformation but also provide high-fidelity stress–strain data for elastoplastic numerical simulations of an FeCrAl alloy. This capability enables accurate thermomechanical response evaluation of FeCrAl alloy cladding.

## 4. Conclusions

The hot compression experiment results demonstrate that FeCrAl alloy exhibits significant nonlinear characteristics over wide temperature and strain rate ranges, with the flow stress decreasing as the temperature increases or the strain rate decreases. The true stress–strain curves under identical thermo-mechanical conditions display a characteristic trend: an initial rapid increase due to work hardening effects, followed by gradual stress reduction after reaching peak stress, governed by thermal softening mechanisms.

Based on the hot compression experimental data, both a strain-compensated Arrhenius constitutive model and an artificial neural network (ANN) model are developed, followed by a comparative error analysis. The ANN model demonstrates superior performance with an AARE of 0.70% and an RMSE of 1.99 MPa, significantly outperforming the strain-compensated Arrhenius constitutive model (AARE = 4.30%, RMSE = 14.47 MPa). These results indicate that the ANN model achieves higher prediction accuracy for flow stress and provides better characterization of the material’s rheological behavior during hot deformation processes.

Finite-element simulations verify the ANN model’s applicability in numerical modeling. The numerical results obtained from ANN-predicted data show strong consistency with the experimental data in both equivalent plastic strain distribution and load–stroke curves. The validated high-fidelity ANN model demonstrates robust reliability in numerical simulations, providing an effective computational tool for assessing the thermomechanical response of FeCrAl alloy cladding components.

## Figures and Tables

**Table 1:** The chemical composition of FeCrAl alloy (wt.%).

C	Si	Mn	P	S	Cr	Nb	Al	Fe
0.020	0.058	0.066	0.200	0.015	23.01	0.045	5.000	Balance

**Table 2:** Coefficients of the equation at different degrees of deformation in a table.

True Strain	$\alpha ({MPa}^{-1})$	$\beta ({MPa}^{-1})$	*n*	*Q* (J/mol)	ln⁡A
0.05	0.00578817	0.0978	11.78032	583,094.175	56.48958
0.10	0.00480784	0.0705	10.39722	585,825.067	56.80832
0.15	0.00434381	0.0659	10.77661	650,994.569	63.27652
0.20	0.00417564	0.0614	10.47091	648,180.364	62.84802
0.25	0.00408467	0.0590	10.27589	648,081.784	62.76339
0.30	0.00403675	0.0577	10.15583	647,236.520	62.65377
0.35	0.00403047	0.0572	10.05910	644,882.840	62.39976
0.40	0.00405917	0.0568	9.906700	638,400.738	61.72825
0.45	0.00411368	0.0565	9.73116	631,297.919	60.99076
0.50	0.00419438	0.0568	9.56919	624,389.253	60.24838
0.55	0.00429911	0.0585	9.60889	6,251,977.097	60.18814
0.60	0.00439022	0.0583	9.29741	6,198,135.363	59.75589
0.65	0.004532131	0.0591	9.08570	6,179,084.598	59.54815
0.70	0.004686720	0.0609	9.03063	6,250,485.214	60.22132

**Table 3:** Comparison of the models’ error depending on the structure of ANN used.

3-m-n-1	*n_v_*	T (min)	R	AARE (%)	RMSE (MPa)	Prod
3-9-4-1	81	78	0.9996	1.22	2.51	2.791
3-10-5-1	101	80	0.9998	0.90	1.93	2.125
3-11-6-1	123	81	0.9998	0.83	1.72	1.905
3-12-8-1	161	82	0.9997	0.70	1.99	2.110

**Table 4:** Prediction errors of the Arrhenius constitutive model and ANN model.

Model	R	AARE (%)	RMSE (MPa)
ANN	0.9995	0.70	1.99
Arrhenius	0.9846	4.30	14.47

**Figure A1:**
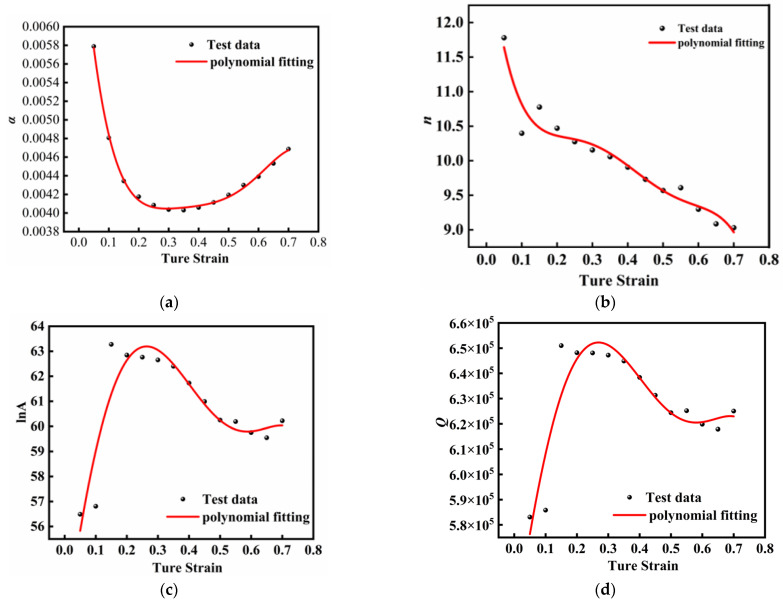
The linear fitting curves of material constants, activation energy with strain obtained through fifth-order polynomial: (**a**) α−ε; (**b**) n−ε; (**c**) ln⁡A−ε; (**d**) Q−ε.

**Figure A2:**
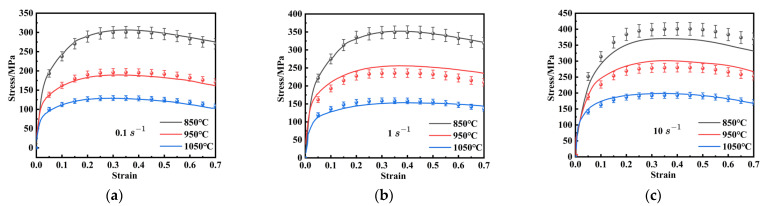
Comparison of experimental flow stress (curve) and predicted flow stress by Arrhenius constitutive model obtained through fifth-order polynomial (symbol): (**a**) 0.1 s^−1^; (**b**) 1 s^−1^; (**c**) 10 s^−1^.

## Data Availability

The original contributions presented in the study are included in the article, further inquiries can be directed to the corresponding author.

## Appendix A

Here, a fifth-order polynomial was utilized to fit the Arrhenius constitutive equation, with the objective of examining the impact of polynomial order on the predictive capacity of the Arrhenius constitutive equation.

Based on the values of ln*A*, *α*, *n*, and *Q* at various true strains, the correlation between these four material parameters and the strain *ε* was established using a fifth-order polynomial fit, with the results depicted in Figure A1 and the corresponding fitting equation presented as Equation (A1).

(A1) $$\left\{\begin{array}{c} lnA=51.82601+86.62086\varepsilon -112.35355\varepsilon^{2}-453.67883\varepsilon^{3}+1146.89539\varepsilon^{4}\\ -696.91612\varepsilon^{5}\\ \alpha =0.00739-0.04138\varepsilon +20121\varepsilon^{2}-0.48146\varepsilon^{3}+0.5654\varepsilon^{4}-\\ 0.25559\varepsilon^{5}\\ n=13.30487-44.71441\varepsilon +263.6326\varepsilon^{2}-741.09778\varepsilon^{3}+957.0178\varepsilon^{4}-\\ 462.92305\varepsilon^{5}\\ Q=537763.73304+817771.47176\varepsilon -668125.13525\varepsilon^{2}-5984787.44146\varepsilon^{3}+\\ 13487440\varepsilon^{4}-8005163.08201\varepsilon^{5} \end{array}\right.$$

Based on this equation, and leveraging this equation, the predicted flow stress values at arbitrary strain levels can be calculated and juxtaposed with the experimental measurements, with the results presented in Figure A2. The error bars in the graph indicate the uncertainty of the Arrhenius model predicted values within a 5% range of the experimental measurements, which facilitates the assessment of the consistency between the model predictions and the experimental measurements.

Additionally, Table A1 presents the values of the three evaluation metrics—R, AARE, and RMSE—obtained using the fifth-order and eighth-order polynomials (the polynomial orders employed in Section 3.2). As evidenced by the values of the evaluation metrics in the table, while the predictive performance of the fifth-order and eighth-order polynomials is highly similar, with identical performance in the AARE metric, the eighth-order polynomial exhibits a slight advantage in both R and RMSE. Consequently, the selection of the eighth-order polynomial for fitting provides superior predictive performance, particularly when a higher degree of fit and predictive accuracy are required.

**Table A1:** Comparison of predictive performance of Arrhenius model with different polynomial orders.

Polynomial	*R* (%)	*AARE* (%)	*RMSE* (MPa^−1^)
5-order	98.47	4.30	14.52
8-order	98.49	4.30	14.47

## Appendix B

The constitutive model of flow stress in the form of hyperbolic sine functions is not readily implementable within the Abaqus/Explicit finite-element framework and necessitates encoding through the VUHARD user subroutine provided, utilizing FORTRAN code. Within the VUHARD subroutine, four variables must be defined: SYIELD, HARD(1), HARD(2), and HARD(3). Here, SYIELD denotes the actual yield stress, while HARD(1), HARD(2), and HARD(3) correspond to the derivatives of the yield stress with respect to strain, strain rate, and temperature, respectively.

Given the infeasibility of physically acquiring training data to evaluate these derivative values during experimental testing, it is imperative to compute the derivatives of the ANN output with respect to the input vector through the internal weights and bias terms of the neural network. The following exposition delineates the acquisition of the pertinent equations that are essential for the construction of the VUHARD subroutine.

First, compute the derivatives of the ANN with respect to the input vectors $\vec{x}=(\varepsilon ,\dot{\;\varepsilon },\;T)$, as shown by the following four equations, where *w*_1_, *b*_1_, *w*_2_ and *b*_2_ are the weights and biases of the network, and $\vec{w}$ is the weight of the output layer.

(A2) $$\vec{z_{1}}=\exp(-w_{1}\;\cdot \;\vec{x}-b_{1})$$

(A3) $$\begin{array}{c} \vec{z_{2}}=\exp(w_{2}\;\cdot \;\vec{z_{1}}+b_{2}) \end{array}$$

(A4) $$\vec{z_{3}}=\vec{w}{}^{\circ}\frac{\vec{z_{2}}}{{\left(1+\vec{z_{2}}\right)}^{2}}$$

(A5) $$\begin{array}{c} \vec{z_{4}}=\frac{\vec{z_{1}}}{{\left(1+\vec{z_{1}}\right)}^{2}} \end{array}$$

Subsequently, we can derive the three partial derivatives of the flow stress with respect to the input triadic vector, as illustrated in Equation (A6). Ultimately, by reversing the normalization operations applied during the processing of the training data, we can compute the three partial derivatives of the flow stress with respect to the input vector, yielding the final equation, (A7), which is utilized for the numerical implementation of the ANN constitutive law within the finite-element code.

(A6) $${\vec{s}}^{\prime }={\vec{w}}_{1}^{T}\cdot [({\vec{w}}_{2}^{T}\cdot \;\vec{z_{3}}){}^{\circ}\vec{z_{4}\;}]$$

(A7) $$\left\{\begin{array}{c} \partial \sigma /\partial \varepsilon^{p}=s_{1}^{\prime }\;\frac{{\left[\sigma \right]}_{max}-{\left[\sigma \right]}_{min}}{{\left[\varepsilon^{p}\right]}_{max}-{\left[\varepsilon^{p}\right]}_{min}}\\ \partial \sigma /\partial \dot{\varepsilon }=\frac{s_{2}^{\prime }}{\dot{\varepsilon }}\;\frac{{\left[\sigma \right]}_{max}-{\left[\sigma \right]}_{min}}{{\left[\dot{\varepsilon }\right]}_{max}-{\left[\dot{\varepsilon }\right]}_{min}}\\ \partial \sigma /\partial T=s_{3}^{\prime }\;\frac{{\left[\sigma \right]}_{max}-{\left[\sigma \right]}_{min}}{{\left[T\right]}_{max}-{\left[T\right]}_{min}} \end{array}\right.$$
